# Identification of the UGT Family and Functional Validation of *MwUGT2* in *Meconopsis wilsonii*

**DOI:** 10.3390/plants14060944

**Published:** 2025-03-17

**Authors:** Lin Zhou, Xiaojuan Chen, Wenkun Su, Zhi Ou, Yan Qu

**Affiliations:** Southwest Research Center for Engineering Technology of Landscape Architecture (State Forestry and Grassland Administration), Yunnan Engineering Research Center for Functional Flower Resources and Industrialization, College of Landscape Architecture and Horticulture Science, Southwest Forestry University, Kunming 650224, China; zhoulincr@126.com (L.Z.); flyersw@163.com (X.C.); swk2644809755@163.com (W.S.)

**Keywords:** Cyanidin-3-O-glucoside, Cyanidin-3-O-sambubioside, flower color, metabolomics, transgenic tobacco

## Abstract

Flower color is one of the most ornamental values of *Meconopsis wilsonii*, but very limited studies have been reported on its flower color formation. The UDP-glycosyltransferase (UGT) gene family plays a crucial role in plant flower color formation. In this study, the full-length transcriptome data of *M. wilsonii* was used to identify MwUGTs, focusing on protein physicochemical properties’ subcellular localization, and phylogenetic relationships. In addition, sequence analysis, expression pattern analysis, subcellular localization, and functional validation of *MwUGT2* were also performed. A total of 26 MwUGTs were identified in full-length transcriptome and clustered into eight subgroups. Phylogenetic analysis and KEGG database annotation showed that *MwUGT2* is associated with anthocyanin synthesis and accumulation. Subsequently, based on the expression of *MwUGT2* during flower development and in different tissues, it was preliminarily determined that MwUGT2 plays a role in the flower bud stage. Subcellular localization assays suggested that *MwUGT2* is present in the nucleus and cytoplasm. Overexpression in *Nicotiana tabacum* showed that *MwUGT2* significantly increased the content of Cyanidin-3-O-glucoside and resulted in dark pink flowers in transgenic plants. In summary, our findings suggest that *MwUGT2* plays a crucial role in the biosynthesis of anthocyanin and will also contribute to understanding the mechanisms of flower color formation in *M. wilsonii*.

## 1. Introduction

Flower color is one of the most important features in ornamental plants, and it is critical for reproductive ecology and species evolution in flowering plants [1]. The development of flower color is related to petal tissue structure, the pH of the vacuole, and the type and content of pigments in the petal cells, which is mainly determined by the accumulation of pigment [2]. The pigments that affect the color of petals are mainly composed of three types: flavonoids, carotenoids, and betaine [3]. Of these compounds, flavonoids are the main flower pigments that confer a wild range of colors to flowers and accumulate in petal vacuoles [4]. Based on their basic skeleton, flavonoids can be classified into flavonols, flavones, flavanols, flavanones, isoflavones, proanthocyanidins, and anthocyanins [5,6]. Among them, anthocyanins are major pigments, that produce various colors from purple to red in flowers [7,8,9]. However, anthocyanins are extremely unstable under natural conditions and exist mainly in the form of glycosylation to form glycosides [10,11]. The glycosidic substances formed not only change the color of anthocyanins but also enhance their stability and biological activities [12,13]. So, glycosylation is crucial for the formation of plant flower color.

Glycosylation is an important modification reaction that plays significant biological roles in plant growth and responses to biotic and abiotic stresses [14], including hormone homeostasis, UV protection, pollination, and defense [15,16,17]. Anthocyanins are usually modified by glycosylation reactions, and result in changes to multiple physiological properties, including stabilization and solubilization. *Anthurium scherzerianum* ‘Alabama’ is known for its bright red, heart-shaped spathe [18]. *A. scherzerianum* ‘Xueyu’, a tissue culture mutant of *A. scherzerianum* ‘Alabama’ with the white spathe [19]. It was shown that the spathes of *A. scherzerianum* ‘Xueyu’ contained almost no anthocyanin and the expression level of the *AnUFGT1* gene was significantly higher in *A. scherzerianum* ‘Alabama’ than in *A. scherzerianum* ‘Xueyu’, suggesting that anthocyanin-deletion mutations in *A. scherzerianum* ‘Xueyu’ are associated with *AnUFGT1* [20]. The overexpression of anthocyanin 3′-O-glucosyltransferase from Gentiana trifloral in *Petunia hybrida* showed an accumulation of blue and purple color anthocyanins in the flower of transgenic *P. hybrida* [21]. Glycosylation is therefore essential for petal coloration.

*Meconopsis* (*Meconopsis* spp.) belongs to the Papaveraceae family and is known as the “Himalayan blue poppy”. Its bright colors and beautiful gestures make it one of the most attractive ornamental flowers in alpine plants [22], but so far, limited information about its flower color formation has been provided. The blue-violet *M. wilsonii* is the model species for the study of the blue flowers of *Meconopsis* but very limited studies have been reported on its flower color formation. The blue-violet mix petal of *M. wilsonii* was mainly complex and formed by Cyanidin and other substances [23]. Further analysis revealed that a total of 27 anthocyanins were detected in the petals of *M. wilsonii*, and all of the anthocyanins were in the form of glycosylation with the glycoylation site of 3-hydroxyl, and the main types of glycosylation are sambubioside, glucoside, and galactoside [24]. Nevertheless, the key enzyme, anthocyanidin 3-O-glycosyltransferase critical for anthocyanin glycosylation, has not been cloned and characterized from *M. wilsonii*.

In the present study, bioinformatics techniques were used to identify UGT family members from the full-length transcriptome of *M. wilsonnii* petals and to analyze physicochemical properties and phylogenetic relationships. *The MwUGT2* gene, which is closely related to anthocyanins, was cloned and transformed into tobacco plants, and their functions in flower color formation were characterized. Functional characterization of the *MwUGT2* gene may help to understand the mechanism of blue flower formation in *Meconopsis* and lay the foundation for future research on blue flower breeding. Our results provide guidance for the investigation of the genetic mechanism of flower color formation in *M. wilsonii*.

## 2. Results

### 2.1. Identification of MwUGTs in M. wilsonii

A total of 26 MwUGTs were identified in the full-length transcriptome of *M. wilsonii*. After that, the *M. wilsonii* UGT family members were named (MwUGT1-MwUGT26) and chemical properties were predicted. The length of the UGT proteins varied from 266 to 549 amino acids, the predicted molecular weight ranged from 61.81 to 60.39 kDa, and the isoelectric point ranged from 4.68 to 6.8. The subcellular localization of these genes indicated that most members were probably in the cell membrane and chloroplasts, while only a few members were in the cytoplasm, nucleus, and peroxisomes (Supplementary Materials Table S1).

### 2.2. Phylogenetic Analysis of MwUGTs

**Figure 1:**
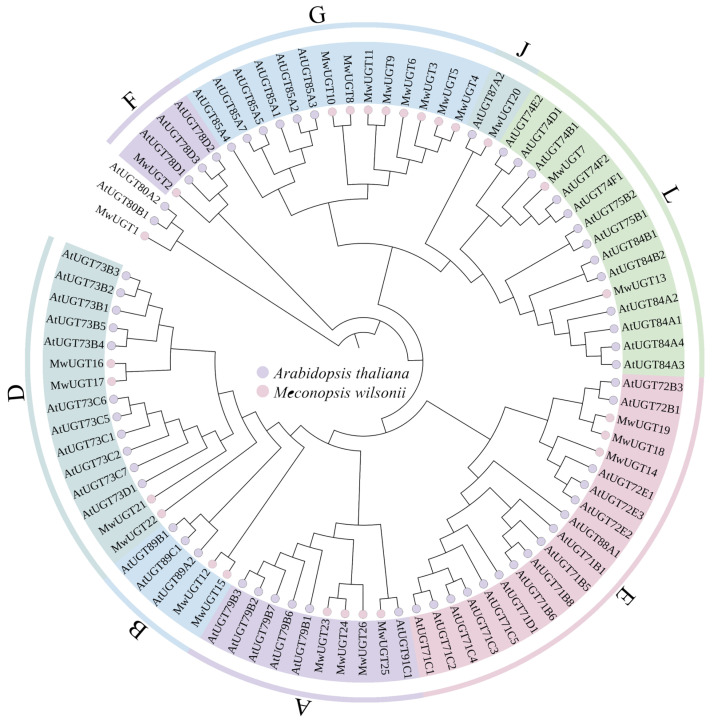
Phylogenetic analysis of the MwUGT proteins among *M. wilsonii* and *A. thaliana*. Different colored strips indicate subfamilies. MwUGTs were divided into A, B, D, E, F, G, J, and L by the known AtUGT proteins.

The phylogenetic analysis of the identified MwUGTs was performed to analyze their grouping pattern and their genetic relationships based on the *A. thaliana* UGT sequences. The *M. wilsonii* UGTs were classified into eight subgroups (Figure 1). The number of UGTs in each group varied: the largest group G had eight UGT members and the smallest group F and J had only one number. MwUGT2 was closely related to anthocyanidin glucosyltransferases AtUGT78D2 from *A. thaliana*. KEGG analyses indicated that MwUGT2 was annotated as anthocyanidin 3-O-glucosyltransferase 7-like (Supplementary Materials Table S2), based on which we hypothesized that MwUGT2 identified in this study might catalyze the last step of anthocyanin biosynthesis.

### 2.3. Cloning and Sequence Analysis of MwUGT2 from M. wilsonii

**Figure 2:**
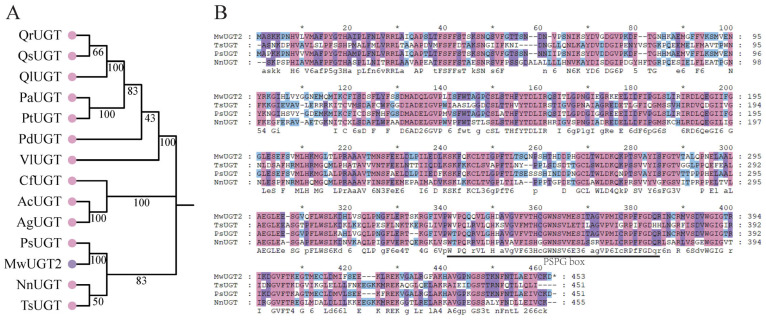
Phylogeny tree and sequence alignment of UGTs from different species. (**A**) Phylogenetic analysis of UGT proteins from different species. GenBank accession numbers are as follows: QrUGT (*Quercus robur* XP_050253999.1), QsUGT (*Quercus suber* XP_023873100.1), QlUGT (*Quercus lobata* XP_030934873.1), PaUGT (*Populus alba* XP_034907570.1), PtUGT (Populus trichocarpa XP_006376354.2), PdUGT (*Paeonia delavayi* AQZ26785.1), VlUGT (*Vitis labrusca* ABR24135.1), CfUGT (*Cornus florida* XP_059636915.1), AcUGT (*Aralia cordata* BAD06514.1), AgUGT (*Apium graveolens* AXU98426.1), PsUGT (*Papaver somniferum* XP_026387438.1), NnUGT (*Nelumbo nucifera* XP_010279580.1), TsUGT (*Telopea speciosissima* XP_043695717.1) (**B**) Amino acid sequences alignment of MwUGT2 protein in *M. wilsonii* with proteins from other species.

In order to further study the MwUGT2 involved in flower color in *M. wilsonii*, we cloned the MwUGT2. Based on sequence information from full-length transcriptome data, the coding region sequences of MwUGT2 were successfully amplified from the flower of *M. wilsonii*. The open reading frame (ORF) of MwUGT was 1362 bp encoding a protein consisting of 453 amino acids, and its theoretical protein molecular weight was 50.11 kDa. Subsequently, the phylogenetic relationships among MwUGT2 and UGTs from diverse of plant species were analyzed, and MwUGT2 was similar to the UGTs from *Papaver somniferum*, *Nelumbo nucifera* and *Telopea speciosissima* (Figure 2A). Further amino acid sequence alignment revealed that MwUGT2 contained the conserved PSPG signature motif in its sequences (Figure 2B).

### 2.4. Analysis of MwUGT2 Expression Patterns and Subcellular Localization

**Figure 3:**
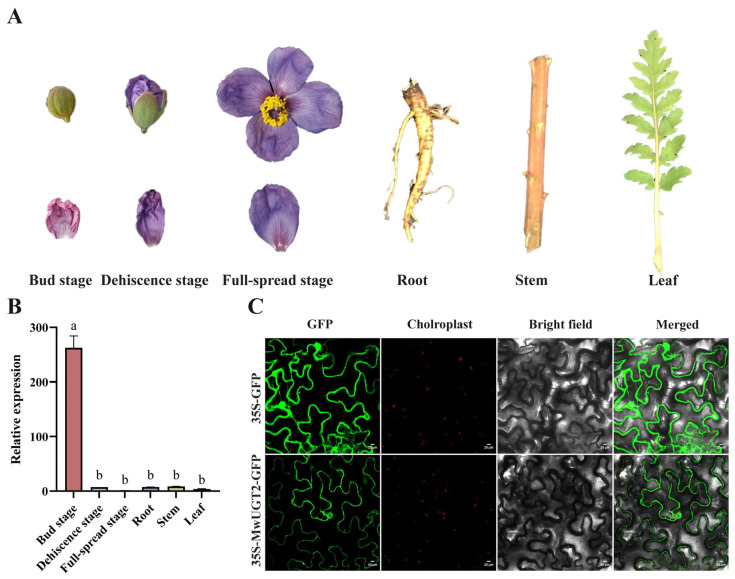
The expression pattern and subcellular localization of *MwUGT2*. (**A**) Phenotypes of the three stages of the flowering process and different tissues. (**B**) Relative expression levels of *MwUGT2* gene in the three stages of the flowering process and different tissues, including bud stage, dehiscence stage, full-spread stage, root stem, and leaf. The expression levels of *MwUGTw2* in the full-spread stage have been arbitrarily set = 1. Error bars indicate standard deviations and different letters above the bars represent significant differences (*p* < 0.05) according to Duncan’s statistical analysis. (**C**) Subcellular localization of MwUGT2-GFP heterologously expressed in *Nicotiana tabacum* leaves. Scale bar, 20 μm.

To examine the relationship between color phenotype and expression levels of *MwUGT2* in *M. wilsonii*, qRT-PCR assays were conducted to analyze the transcript levels of *MwUGT2* in different organs (root, stem, leaf, and flower) and flower development stage (bud stage, dehiscence stage, and full-spread stage). The results obtained demonstrated that *MwUGT2* was expressed in all of the tested organs, with the highest and lowest expression levels in the bud stage and full-bloom stage, respectively (Figure 3A,B). Indicating that *MwUGT2* may play a role in the formation of flower color in *M. wilsonii*, especially during the bud stage.

The subcellular localization of MwUGT2 was examined by analyzing the intracellular localization patterns of fluorescence from their GFP-chimeric proteins in tobacco cells. GFP, controlled by the 35 s promoter, was transformed into tobacco as a control. The green GFP signal was distributed throughout the entire cell (Figure 3C). This indicated that MwUGT2 was located in the nucleus and cytoplasm.

### 2.5. MwUGT2 Promotes Anthocyanin Accumulation in Transgenic Tobacco

**Figure 4:**
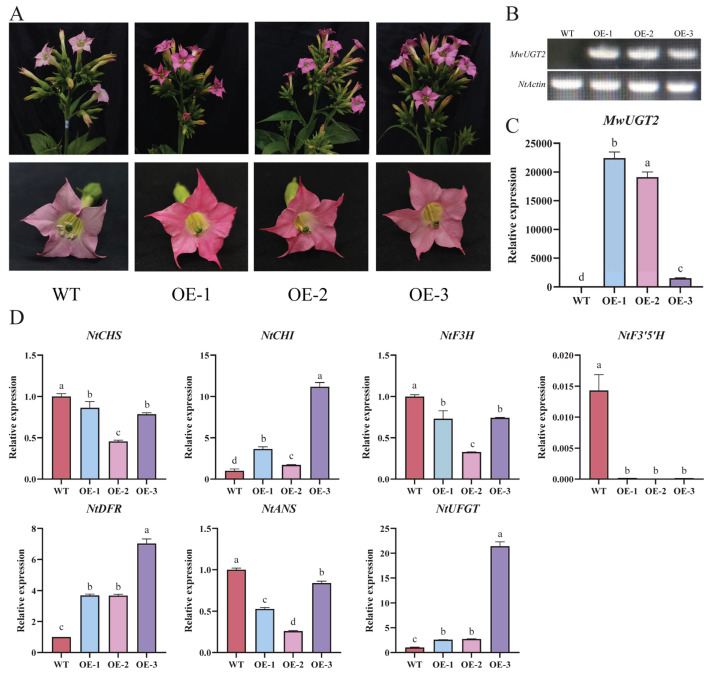
Overexpression of *MwUGT2* contributes to petal discoloration in transgenic tobacco lines. (**A**) Tobacco flowers of wide-type and transgenic lines. WT, wild-type; OE, overexpression. (**B**) Expression profiles of *MwUGT2* in transgenic tobacco flowers. (**C**) The expression of *MwUGT2* in transgenic lines and WT. The expression level of MwUGT2 in WT has been arbitrarily set = 1. Error bars indicate standard deviations and different letters above the bars represent significant differences (*p* < 0.05) according to Duncan’s statistical analysis, (**D**) Analogous. (**D**) The expression patterns of *NtCHS*, *NtCHI*, *NtF3H*, *NtF3′5′H*, *NtDFR*, *NtANS* and *NtUFGT* in WT and transgenic plants. The expression level of *NtCHS*, *NtCHI*, *NtF3H*, *NtF3′5′H*, *NtDFR*, *NtANS* and *NtUFGT* in WT have been arbitrary set = 1, respectively.

To verify the function of *MwUGT2*, *MwUGT2* was overexpressed in tobacco. Compared with the WT, the flowers of *MwUGT2* transgenic plants with visibly increased color. We selected three lines (lines 1, 2, and 3) with the darkest petal color for subsequent experiments (Figure 4A). The petals of *MwUGT2* transgenic tobacco were collected and validated by PCR and qRT-PCR, and three transgenic lines displayed significantly higher *MwUGT2* expression levels (Figure 4B,C). Additionally, the expression levels of the anthocyanin biosynthesis-related genes *NtCHI*, *NtDFR*, and *NtUFGT* were significantly upregulated in MwUGT2-overexpressing flowers, while *NtF3′5′* was significantly down-regulated (Figure 4D).

**Figure 5:**
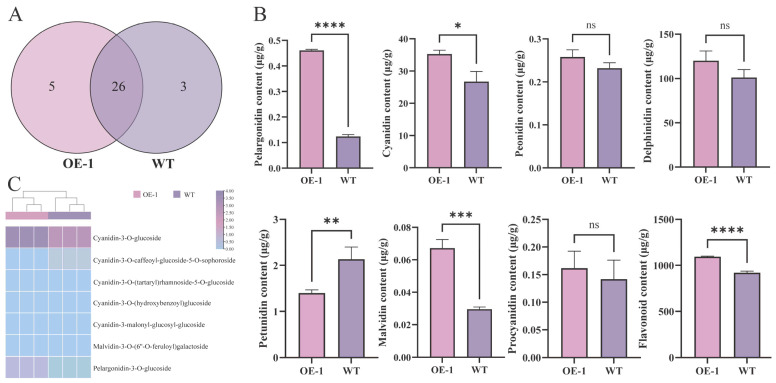
Effect of MwUGT2 on anthocyanin accumulation in transgenic tobacco flower. (**A**) Quantity of anthocyanin metabolites in WT and transgenic tobacco petals. (**B**) The content of Pelargonidin, Cyanidin, Peonidin, Delphinidin, Petunidin, Malvidin, Procyanidin, and Flavonoid in flowers of WT and transgenic plants. Statistical significance was determined using Student’s *t*-test (* *p* < 0.05, ** *p* < 0.01, *** *p* < 0.001, **** *p* < 0.0001, ns indicates not significant). (**C**) Heat map of anthocyanin differential metabolites.

A total of 34 metabolites were identified in the flowers of the *MwUGT2* transgenic and WT K326 tobacco plants (Figure 5A). The results of the secondary classification of anthocyanins showed that Pelargonidin, Cyanidin, Malvidin, and Flavonoid were significantly higher at OE-1 than at WT; Petunidin was significantly lower than at WT (Figure 5B). Further analysis of the above differential metabolites revealed that Cyanidin-3-O-glucoside was the most abundant and most variable differential metabolite in OE-1 and WT (Figure 5C).

## 3. Discussion

Flower color, one of the most important economic traits in ornamental plants, is mainly determined by the types and levels of anthocyanins [25,26]. The glycosylation reaction is the last step in the biosynthesis of many secondary metabolites and also represents a common modification of plant secondary metabolites [27]. The UGT gene is the key enzyme involved in promoting anthocyanin accumulation through glycosylation downstream of the anthocyanin synthesis pathway. However, the mechanism of UGT anthocyanin in *M. wilsonii* was unclear, necessitating its study using the existing technique. In this study, we first analyzed the UGT family of *M. wilsonii*, and according to phylogenetic analysis and KEGG database annotation, the UGT associated with anthocyanin synthesis was screened. Furthermore, we conducted the functional characterization of *MwUGT2* to elucidate the blue-violet flower formation, which will benefit breeding by increasing ornamental value and enrich the understanding of anthocyanin patterning in angiosperms.

In this study, a total of 26 MwUGTs were identified in *M. wilsonii* using bioinformatics methods. To further understand the function of MwUGTs, we constructed a phylogenetic tree of *A. thaliana* and *M. wilsonii* UGTs following a clustering approach. Twenty-six MwUGTs were distributed in eight subgroups, with up to eight members of subclade G. G-group members from *A. thaliana* usually show a specific numerical preference for substrates such as monoterpenes, diterpenes, and deacylated carotenoids [28,29]; differences in substrate choice among G-group members in *C. sinensis* lead to functional diversification and these members influence tolerance to abiotic stresses such as low temperature [30]. Therefore, we hypothesize that members of the G subfamily of *M. wilsonii* may function in protection against cold stress and terpene synthesis. Previous studies exhibited that *AtUGT78D2* in *A. thaliana* is involved in the biosynthesis of anthocyanins [31]. In this study, *MwUGT2* clustered with *AtUGT78D2* from *A. thaliana*. At the same time, KEGG analyses showed *MwUGT2* was annotated as anthocyanidin 3-O-glucosyltransferase 7-like. At the same time, multiple sequence alignment showed that *MwUGT2* carried the typical PSPG sequence motif at the C terminal end, and their final amino acid residue within this motif was histidine [32,33], which means that *MwUGT2* are more likely to be galactosyltransferases. *MwUGT2* was preliminarily defined as the pivotal UGT gene for anthocyanin formation in *M. wilsonii* flowers. Expression pattern analysis showed that *MwUGT2* was expressed in all tissue fractions, with expression in the bud stage shown to be higher than in other periods and tissue fractions. This suggests that *MwUGT2* mainly functions in the bud stage of *M. wilsonii*. The glycosylation reaction of flavonoids catalyzed by UGT mainly occurs in the cytoplasm [34]. Subcellular localization analysis showed that *MwUGT2* is located in the nucleus and cytoplasm. Compared to the wild type, transgenic tobacco plants harboring the *MwUGT2* produced dark pink flowers, and this similar phenomenon leading to additional accumulation of anthocyanins had also been observed in *Rhododendron delavayi* [35], *Nelumbo nucifera* [36], and *Paeonia suffruticosa* [37]. F3′5′H is a key enzyme in the biosynthesis of delphinidin-like anthocyanins, which are normally required for purple or blue flowers [38,39,40], and the introduction of *OhF3′5′* and *CtA3′5′GT* into *Chrysanthemum morifolium* promotes the accumulation of delphinium and its derivatives in chrysanthemum morifolium, which results in the change in the flower color from reddish-purple to violet [41]. In this study, *NtF3′5′H* was significantly downregulated in transgenic tobacco petals, suggesting that *MwUGT2* may inhibit the expression of *NtF3′5′H* and reduce the synthesis of delphinidin and its derivatives. The downregulation of *NtF3′5′H* in transgenic petals could result from metabolic feedback regulation. In studies of *Vitis vinifera* anthocyanins, it was found that, when *V. vinifera* were exposed to light, *VvHY5* activated *VvMYBA1*, which induced the expression of *VvUFGT*, leading to increased anthocyanin biosynthesis [42]. At the same time, *VvMYBA1* induced the expression of *VvBBX44*. When the anthocyanin concentration reached a certain level, it activated *VvBBX44* expression. Then, *VvBBX44* directly repressed the expression of *VvMYBA1* and *VvHY5*, leading to a decrease in *VvUFGT* expression and dynamic equilibrium in anthocyanin concentration [43,44]. Therefore, we hypothesize that rapid glycosylation of anthocyanin by *MwUGT2* may lead to a rapid increase in anthocyanin concentration to a certain level, which activates the expression of transcription factors, and the activated transcription factors inhibit the expression of *NtF3′5′H,* leading to a decrease in *NtF3′5′H* to expression and dynamic equilibrium of anthocyanin concentration. However, pigment analysis revealed an increase in the levels of all pigments except Petunidin. In *Freesia hybrida*, the *Fh3Gt1* gene promotes the glycosylation of a wide range of anthocyanins [45]. The apparent increase in total delphinidin (despite *NtF3′5′H* suppression) might reflect the enhanced stability of its glycosylated forms, as glycosylation protects anthocyanins from degradation. In addition to the significant increase in Cyanidin in the present study, there was also a significant increase in pelargonidin and Petunidin, which may be related to the broad substrate specificity of glycosyltransferases. In *A. thaliana*, *TcCGT1* effectively and regionally catalyzes the 8-C-glycosylation of 36 flavonoids and other flavonoids [46], and we therefore hypothesized that *MwUGT2* has the same function. Further analysis of anthocyanin metabolites in WT and transgenic tobacco corollas showed that Cyanidin-3-O-glucoside was the most abundant and variable differential metabolite in transgenic tobacco and WT.

**Figure 6:**
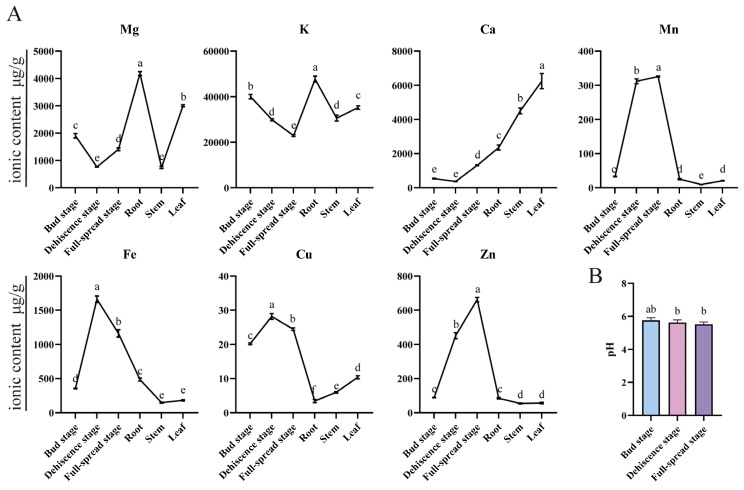
*M. wilsonii* metal ions and pH. (**A**) Metal ion content in different developmental stages and different tissue parts of *M. wilsonii* flowers. Error bars indicate standard deviations and different letters above the bars represent significant differences (*p* < 0.05) according to Duncan’s statistical analysis, Figure 6B Analogous. (**B**) pH values for different developmental stages of *M. wilsonii* flowers.

In this study, transgenic tobacco flower failed show to develop blue-violet coloration. To investigate the phenomenon, we conducted a comparative metabolomic analysis of anthocyanins in *M. wilsonii*. The result showed that Cyanidin-type anthocyanins predominated in *M. wilsonii*, with Cyanidin-3-O-sambubioside (199.38 mg/g, 86.32% of total anthocyanin glycosides) being the most abundant pigment (Supplementary Table S3). Notably, Cyanidin-3-O-sambubioside is critical for blue hues in plants such as *Corydalis ambigua*, where its chelation with Fe^3+^ under pH 6.5 replicates the blue coloration observed in natural petals [47]. In contrast, transgenic tobacco flowers predominantly accumulated Cyanidin-3-O-glucoside, while Cyanidin-3-O-sambubioside was undetectable—consistent with previous reports on tobacco’s limited capacity to synthesize sambubioside derivatives [48]. This suggests that the absence or low activity of the sambubioside-specific glycosyl-transferase in tobacco redirects Cyanidin flux toward glucoside formation, preventing the synthesis of Cyanidin-3-O-sambubioside required for blue pigmentation. In nature, blue flowers commonly contain Delphinidin derivatives, although there are a few exceptions where Cyanidin–metal chelation generates blue hues [49,50,51]. Studies on blue flowers of *Meconopsis* show that Cyanidin derivatives, Fe^3+^, Mg^2+^, and flavonols are essential for the formation of sky blue-color petals in *M. grandis* [52]. In *M. wilsonii*, metabolomic and ionomic analyses revealed that petals at the dehiscence and full-spread stages accumulated significantly higher levels of Mn, Fe, Cu, and Zn compared to other tissues or developmental phases (Figure 6A), while maintaining stable pH across all stages (Figure 6B). Based on these findings, we hypothesize that the blue coloration in *M. wilsonii* flowers is mediated by Cyanidin–metal complexes, facilitated by the coordinated increase in transition metal ions during late petal development.

**Figure 7:**
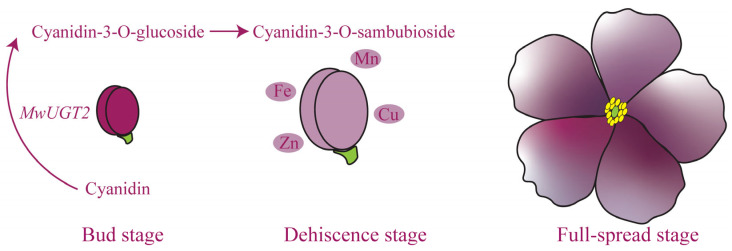
Schematic diagram of the formation of blue-violet flowers of *M. wilsonii*.

In summary, we have predicted a mechanism for the formation of blue-violet flowers in *M. wilsonii* (Figure 7). High expression of *MwUGT2* during the bud stage of *M. wilsonii* promotes the accumulation of Cyanidin-3-O-glucoside, which is processed into Cyanidin-3-O-sambubioside at the dehiscence stage and complexes with metal ions to form complexes.

## 4. Materials and Methods

### 4.1. Sample Collection

*Meconopsis wilsonnii* Grey-Wilson was collected from Palanquin Snow Mountain, Yunnan Province, China (102°50′5″ E, 26°5′1″ N, 3500 m). The roots, stems, leaves, and flowers (bud stage, dehiscence stage, and full-bloom stage) were collected. Transgenic and Wide Type (WT) tobacco lines were bred at the Nursery Base of Southwest Forestry University in Kunming, Yunnan Province, China (102°45′53″ E, 25°4′0″ N) and corollas were collected at full bloom. All samples mentioned above were frozen immediately in liquid nitrogen and then stored at −80 °C for further use.

### 4.2. Identification of MwUGTs

The sequence information of *M. wilsonnii* was obtained from the full-length transcriptome of *M. wilsonnii* petals obtained by the group in the early stage (PRJNA1095676). The Hidden Markov Model (HMM) profile corresponding to the UGT structural domain (PF00201) was downloaded from InterPro (https://www.ebi.ac.uk/interpro/entry/pfam, accessed on 20 December 2024) and then screened for conserved sequences (E-value < 1 × 10^−5^) among all *M. wilsonii* protein sequences using the Simple HMM Search function of the TBtools 1.1 software. Furthermore, the CDD function of the NCBI website and SMART online were employed to analyze and integrate the obtained protein sequences. Erroneous sequences were manually removed, ensuring the presence of UDP-glycosyltransferase structural domains. Ultimately, sequences containing the complete UGT structural domain were selected. The online software ExPASy (http://web.expasy.org/protparam, accessed on 20 December 2024) was used to analyze the physicochemical properties of screened UGT. Subcellular localization was predicted using the online software Cell-PLoc 2.0 (http://www.csbio.sjtu.edu.cn/bioinf/plant-multi, accessed on 21 December 2024). The secondary structures of the proteins were predicted using the online software SOPMA (http://npsa-prabi.ibcp.fr/cgi-bin/npsa_automat.pl?page=npsa_sopma.html, accessed on 21 December 2024).

### 4.3. Phylogenetic Analysis of MwUGTs

For phylogenetic analysis, the full-length amino acid sequences of UGT proteins from *A. thaliana* and *M. wilsonnii* were aligned by MEGA 11.0.13 software and was used to build the tree using the neighbor-joining method (Bootstrap value was set to 1000). Arabidopsis UGT protein sequences were retrieved from TAIR (https://www.arabidopsis.org/, accessed on 22 December 2024). The online software iTOL (https://itol.embl.de/, accessed on 22 December 2024) was used to modify the tree.

### 4.4. Cloning MwUGT2

Total RNA was isolated from the flower using Omega Plant RNA Kit (Omega, MA, USA), following the manufacturer’s instructions. First-strand cDNA was synthesized using 5 All-In-One RT MasterMix (ABM, Shanghai, China), according to the manufacturer’s protocol. Full-length sequences of MwUGT2 open reading frames were obtained from the database and confirmed by isolating the sequence from flowers using MwUGT2-F (ATGGCATCAAAAAAGCCAAACC) and MwUGT2-R (TCAGTCTTTACAAACTATCTCTGCC) primers. PCR amplification was conducted by Green Tap Mix (Vazyme, Nanjing, China). The correct bands from agarose gel electrophoresis were recovered and then connected with the pMD19-T vector (TaKARa, Beijing, China) for sequencing. After alignment, the sample showed a consistent sequence with that from the full-length transcriptome of *M. wilsonnii* petals was used for the next experiments.

The amino acid sequences encoded by MwUGT2 and their homologs from other species, obtained using BLAST searches, were used to construct phylogenetic trees based on the neighbor-joining method in MEGA software, setting up bootstrap to test 1000 repetitions.

### 4.5. Construction of Overexpression Vectors

Construct pBWA (V)HS-MwUGT2-Glosgfp vectors (completed by Wuhan Boyuan Biotechnology Co., Ltd., Wuhan, China). The vector was digested using the restriction enzyme Bsal, and the vector digest was purified with a PCR purification kit and ligated with the PCR product. Transform 10 μL of ligation product into DH5α *E. coli* competent cells. The transformed cells were evenly coated on Petri dishes containing Kan resistance and cultured at 37 °C. After 12 h, 10 plaques were picked for simultaneous plaque PCR identification. Select the bacterial liquid corresponding to 1–3 positive bands, take 100 μL for sequencing, and inoculate the remaining 400 μL bacterial liquid into LB medium containing 10 mL Kan resistance and shake the bacteria. After the sequencing results are available, select the medium with the correct sequencing to extract the plasmid.

### 4.6. Agrobacterium-Mediated Infiltration Tobacco

Agrobacterium-mediated tobacco transformation was performed as previously described by Huazhong Agricultural University [53]. Genomic DNA of transgenic tobacco lines was isolated to confirm the presence of transgenes by using the OE-MwUGT2-F(ATTGACCGATTCCTTGCGGT) and OE-MwUGT2-R(GAGGGCGTGGATATGTCCTG) primer pairs.

### 4.7. Quantitative Real-Time PCR Analysis

Total RNA was isolated from the roots, stems, leaves, and flowers (bud stage, dehiscence stage, and full-bloom stage) of *M. wilsonnii* and flowers of transgenic tobacco lines, and first-strand cDNA was synthesized. Primers used in the qPCR assay were designed through Primer-BLAST (https://www.ncbi.nlm.nih.gov/tools/primer-blast/index.cgi?LINK_LOC=BlastHome, accessed on 20 January 2025) and are present in Table S4. Then, the qPCR reactions were recommended by the Light Cycler 480 II (Hoffmann-La Roche, Basel, Switzerland). *MwActin* and *NtActin* as internal controls for *M. wilsonnii* and tobacco, respectively. The relative expression levels of related genes were normalized to the relative expression level of *MwActin* or *NtActin*, using the 2^−ΔΔCt^ method. The experiment included three biological replicates.

### 4.8. Targeted Metabolome Testing and Analysis

The petals were sent to Wuhan Meitville Biotechnology Co., Ltd. (Wuhan, China) for anthocyanin-targeted metabolome assay, with three biological replicates for each sample, which were detected by liquid chromatography-mass spectrometry (LC-MS/MS) technique.

Anthocyanins were tested and analyzed; fresh petal samples were freeze-dried and then ground (30 Hz, 1.5 min) to powder form using a ball mill. Then, 50 mg of the powder was weighed and dissolved in 500 μL of extract (50% aqueous methanol containing 0.1% hydrochloric acid), vortexed for 5 min, ultrasonicated for 3 min, and centrifuged for 3 min at 4 °C and 12,000 r min^−1^. The supernatant was aspirated, repeated once, combined, filtered through a 0.22 μm filter membrane, and stored in a feed bottle.

Ultra-performance liquid chromatography (UPLC) and Tandem Mass Spectrometry (MS/MS) were used for data acquisition: the column was ACQUITY BEH C18; the flow rate was 0.35 mL/min; the injection volume was 2 μL. The sample volume was 2 μL. A 0.1% aqueous formic acid solution (A) and a 0.1% methanol solution of formic acid (B) were used as mobile phases 0 min The proportion of phase B was 5%, which was increased to 50% at 6.00 min, to 95% at 12.00 min, and held for 2 min, and then decreased to 5% at 14 min, and equilibrated for 2 min. Mass spectrometry conditions: Electrospray Ionization (ESI) at 550 °C, Mass Spectrometry Voltage (MSV) at 5500 V in Positive Ion Mode (PIM), Curtain Gas (CUR) 35 psi. In Q-Trap6500+, each ion pair was scanned and detected according to the Declustering Potential (DP) and Collision Energy (CE). Both qualitative and quantitative anthocyanins were constructed based on the standards to build a Metware Database (MWDB) database, the data detected by mass spectrometry were qualitatively analyzed, and the mass spectrometry data were processed by Analyst 1.6.3 software.

### 4.9. Statistical Analysis

The variable importance in projection (VIP) value was calculated using partial least squares discriminant analysis (PLS-DA). Metabolites were considered differentially changed between two groups if the VIP ≥ 1, and fold change ≥2 or ≤0.5. For the analysis of qPCR and Metal ion content, experimental data were represented as mean values ± SD from three biological replicates. Statistical significance was evaluated by the Duncan statistical analysis (*p* < 0.05).

## 5. Conclusions

In this paper, the UGT family of *M. wilsonii* was identified, and the anthocyanin synthesis-related UGTs were cloned and functionally verified. Twenty-six MwUGTs were identified and divided into eight groups. Phylogenetic analyses and KEGG database annotations indicate that *MwUGT2* is associated with anthocyanins. Amino acid sequence comparison analysis showed that *MwUGT2* contains a glycosyltransferase structural domain and is attributed to 3GT, suggesting that *MwUGT2* may process anthocyanins. Expression pattern analysis showed that *MwUGT2* was highly expressed during the bud stage of *M. wilsonii*, suggesting that it may act mainly during the bud stage. Subcellular localization revealed that *MwUGT2* is located in the nucleus and cytoplasm. Transgenic tobacco phenotypic and physiological results showed that overexpression of *MwUGT2* showed a dark pink color in flowers of all transgenic lines due to Cyanidin-3-O-glucoside accumulation. This work may inspire further research on the molecular mechanisms of UGTs in *M. wilsonii*.

## Data Availability

The raw sequence data are available at the NCBI Sequence Read Archive: PRJNA1095676.

## Supplementary Materials

The following supporting information can be downloaded at: https://www.mdpi.com/article/10.3390/plants14060944/s1, Table S1: MwUGTs Protein Physicochemical Properties; Table S2: MwUGTs KEGG Functional Notes; Table S3: Anthocyanin metabolites of *Meconopsis Wilsonnii* petals; Table S4: Real-time fluorescent quantitative primer.
